# Insulin Resistance as a Dynamic Correlate of Fibrosis Status in Chronic Hepatitis B: A Visit-Level Longitudinal Risk Stratification Framework

**DOI:** 10.3390/life16060939

**Published:** 2026-06-02

**Authors:** Abdalwahab Omar Alshammari, Idris Adewale Ahmed

**Affiliations:** 1 School of Applied Science, Lincoln University College, Petaling Jaya 47301, Selangor, Malaysia; 2 Hail Health Cluster, Hail 2440, Saudi Arabia

**Keywords:** hepatitis B, chronic, insulin resistance, liver fibrosis, metabolic syndrome, longitudinal studies

## Abstract

Background: Viral replication is a major factor in chronic hepatitis B (CHB). Still, the extent to which host metabolic dysfunction contributes to fibrosis risk remains unclear, particularly in studies that follow patients over time. Because most research relies solely on baseline assessments, it may overlook how metabolic changes and fibrosis interact as the disease progresses. Methods: We conducted a retrospective longitudinal cohort study of 304 adults with CHB using electronic medical records collected across 4 visits over 18 months. Repeated metabolic parameters and non-invasive fibrosis indices were examined using population-averaged and mixed-effects models. The associations we observed represent time-specific co-variation between exposures and outcomes measured at the same time point, rather than earlier predictors of later outcomes. Results: Across 1216 person-visits, 421 visit-level fibrosis risk events were recorded (34.6%). Incident clustered metabolic abnormalities occurred at a rate of 21.43 per 100 person-years. Among the metabolic syndrome components, insulin resistance showed the most consistent independent association with visit-level fibrosis risk status. In contrast, after adjustment, longitudinal trends in BMI, lipid measures, and transaminases did not independently distinguish patients with fibrotic progression. A practical clinical model based on age, AST, platelet count, and fasting glucose demonstrated moderate discrimination across risk strata (AUC = 0.772). Conclusions: In CHB, insulin resistance is consistently linked to visit-level fibrosis risk status. Longitudinal metabolic monitoring using routine clinical data provides a practical, scalable way to assess fibrosis risk, especially in resource-limited settings. These findings support incorporating time-based metabolic assessment into CHB care pathways alongside virological factors.

## 1. Introduction

Chronic hepatitis B virus (HBV) infection affects nearly 300 million individuals globally and remains a leading cause of cirrhosis and hepatocellular carcinoma-related mortality [1]. Fibrosis progression represents the central pathological process underlying these outcomes, yet substantial inter-individual heterogeneity persists even among patients with comparable virological profiles [2]. Beyond viral replication and host immune responses, accumulating evidence implicates host metabolic dysfunction as a correlate of liver injury within a broader biological system context [3,4].

Insulin resistance (IR) is prevalent in chronic HBV and has been linked to steatosis, impaired antiviral response, and cardiometabolic risk [5]. However, its role in HBV-related fibrogenesis remains incompletely defined. Most available studies are cross-sectional, limiting inference on temporality and obscuring whether IR precedes fibrosis progression or reflects secondary metabolic perturbation [5].

Evidence for other metabolic syndrome components in HBV is inconsistent. Unlike non-alcoholic fatty liver disease (NAFLD), where body mass index (BMI), dyslipidemia, and IR closely track fibrosis risk, HBV studies report weak or null associations between BMI, lipid fractions, and fibrosis severity [6,7]. In some cohorts, steatosis and higher BMI coexist with milder fibrosis after accounting for viral factors [8], suggesting that systemic metabolic markers may inadequately reflect intrahepatic metabolic stress relevant to fibrogenesis.

A key limitation of existing literature is reliance on static baseline measurements. Fibrosis progression is inherently dynamic, with metabolic exposures fluctuating over time. Longitudinal analyses in metabolic liver disease indicate that time-updated IR may capture fibrosis risk more informatively than static anthropometric measures, highlighting the value of repeated metabolic assessment in fibrosis risk evaluation [9,10,11]. Table S1 summarizes the methodological approaches of prior studies examining metabolic factors in CHB fibrosis, highlighting that most employed cross-sectional designs or baseline-only analyses. Only two prior studies incorporated repeated metabolic measures, and none utilized time-updated HOMA-IR with population-averaged models to capture within-individual variation. The present study addresses this gap by employing a longitudinal, time-resolved analytic framework that examines contemporaneous associations between dynamic metabolic exposures and fibrosis status.

From a translational perspective, scalable fibrosis risk stratification remains a priority, particularly in resource-limited settings where elastography and advanced imaging are not widely accessible. Longitudinal approaches leveraging routinely collected laboratory data offer a pragmatic pathway to risk stratification [12,13,14]. This gap highlights the need for time-resolved analyses that integrate metabolic dynamics with fibrosis progression within a unified biological framework.

We therefore conducted a retrospective longitudinal cohort study of patients with chronic HBV integrating repeated metabolic assessments with non-invasive fibrosis outcomes. The objectives were to: (i) evaluate whether insulin resistance independently associates with contemporaneous fibrosis status; (ii) clarify the contributions of BMI and lipid parameters; and (iii) develop a visit-level, resource-efficient risk stratification framework. By aligning time-updated metabolic measures with contemporaneous fibrosis assessments, this study interrogates time-resolved metabolic-fibrotic interactions in chronic HBV. Importantly, observed associations reflect visit-level biological interactions rather than prospective time-lagged prediction, and all causal claims are explicitly avoided throughout.

## 2. Materials and Methods

**Study Design, Setting, and Participants**

This retrospective longitudinal cohort study evaluated associations between metabolic dysfunction, particularly insulin resistance, and visit-level fibrosis risk status in chronic HBV within a time-resolved analytical framework. Although data were collected longitudinally, metabolic exposures and fibrosis status were analyzed contemporaneously at each visit without temporal lag (e.g., exposure at visit t − 1 predicting outcome at visit t). Accordingly, findings reflect visit-level biological interactions rather than prospective time-lagged prediction. The study was conducted across three tertiary hospitals in Hail, Saudi Arabia, between January 2022 and December 2024. Data were extracted from electronic medical records and laboratory systems. Adults (≥18 years) with confirmed chronic HBV (HBsAg ≥ 6 months) and available longitudinal metabolic and fibrosis data were eligible. Visits were included in modeling analyses if exposure, outcome, and covariate data were available; visits with missing key variables were excluded from regression analyses.

**Eligibility Criteria**

**Inclusion**: Chronic HBV; baseline and follow-up fasting glucose and insulin; baseline and ≥1 follow-up fibrosis assessment; absence of HCV, HIV, or other chronic liver diseases.

**Exclusion**: Pregnancy; long-term systemic corticosteroid use. Patients with advanced fibrosis at baseline were excluded from incident analyses but retained for descriptive characterization.

**Exposure Variables**

The primary exposure was insulin resistance estimated using the homeostatic model assessment:

$$\mathrm{HOMA}\text{-}\mathrm{IR}=\frac{\mathrm{fasting}\;\mathrm{insulin}\;(\mathrm{µIU}/\mathrm{mL})\times \mathrm{fasting}\;\mathrm{glucose}\;(\mathrm{mmol}/L)}{22.5}$$

Secondary metabolic variables included body mass index (BMI), fasting glucose, triglycerides, high-density lipoprotein (HDL), and low-density lipoprotein (LDL).

**Outcome Definition**

The primary outcome was a visit-level fibrosis risk event, defined using a hierarchical combination of imaging-confirmed progression, APRI/FIB-4 threshold transitions, and documented clinical cirrhosis using the following criteria:

***Imaging-Confirmed Progression (where available):***

Transient elastography (FibroScan) increases from baseline: progression from F0–F1 to F2 or higher, or an increase of ≥1 stage with the final stage ≥ F2. Ultrasound-confirmed progression to cirrhosis or portal hypertension.

***APRI (AST-to-Platelet Ratio Index) Thresholds:***

APRI = [AST (U/L)/ULN AST] × 100/Platelet count (10^9^/L).

Threshold for significant fibrosis (≥F2) is APRI > 0.7 (sensitivity: 77%, specificity: 72%). Threshold for advanced fibrosis/cirrhosis (F3–F4) is APRI > 1.0 (sensitivity: 61%, specificity: 83%) [15].

***FIB-4 Thresholds:***

FIB-4 = [Age (years) × AST (U/L)]/[Platelet count (10^9^/L) × √ALT (U/L)].

Threshold for significant fibrosis (≥F2) is FIB-4 > 1.45 (sensitivity: 74%, specificity: 68%). Threshold for advanced fibrosis/cirrhosis (F3–F4) is FIB-4 > 3.25 (sensitivity: 67%, specificity: 85%) [14].

***Event Definition***

An index-defined fibrosis risk event was recorded if any of the following occurred between consecutive visits:

(a) Imaging-defined progression (as above);
(b) Crossing of APRI threshold from below to above 0.7 (or above 1.0 for advanced fibrosis);
(c) Crossing of FIB-4 threshold from below to above 1.45 (or above 3.25 for advanced fibrosis);
(d) Clinical diagnosis of cirrhosis documented in medical records.

***Important Methodological Note***

Because APRI and FIB-4 incorporate age, AST, and platelet count, predictors in the visit-level model partially overlap with outcome components. Accordingly, the model is specified as a pragmatic clinical triage framework rather than an estimator of independent structural fibrosis progression, and performance metrics are interpreted within this context. Across the analytic dataset, 421 fibrosis progression events were observed among 1216 visits.

**Covariates**

Covariates included age, sex, HBV DNA level, antiviral therapy status, diabetes, and hypertension.

**Statistical Analysis**

Time-updated metabolic variables were modeled as contemporaneous covariates measured at the same visit as fibrosis status. Population-averaged associations were estimated using generalized estimating equations (GEE), and subject-specific effects were evaluated using mixed-effects models with patient-level random intercepts. A visit-level clinical utility model was developed using multivariable logistic regression. Model covariates were prespecified based on clinical relevance and prior literature, and all candidate predictors were entered simultaneously without automated variable selection to reduce overfitting and preserve interpretability. Internal validation was performed using 5-fold grouped cross-validation, ensuring that all repeated visits from a single patient were assigned to the same fold to prevent information leakage. Using R 4.2.2, Bootstrap resampling with 2000 replicates was additionally used to estimate optimism in model discrimination. Model performance was assessed using receiver operating characteristic (ROC) curves and area under the curve (AUC), with both apparent and optimism-corrected estimates reported.

**Missing Data, Time-Lag Sensitivity, and Mathematical Coupling**

Missing data were addressed. Patients with incomplete longitudinal records or irregular follow-up were excluded during cohort construction, resulting in a final analytical dataset with no missing values for any variable required in the statistical models. A time-lag sensitivity analysis was conducted using GEE with HOMA-IR measured at the preceding visit (t − 1, six months prior) as an antecedent exposure associated with fibrosis status at the current visit (t), separate from the primary contemporaneous analysis. This analysis yielded an attenuated but directionally consistent estimate (OR = 1.028, 95% CI: 1.015–1.041, *p* < 0.001) compared to the primary contemporaneous association.

**Reporting and Ethics**

The study was reported in accordance with STROBE guidelines and aligned, where applicable, aligned with TRIPOD recommendations for reporting the model development component. Ethical approval was obtained, and all data were de-identified prior to analysis.

## 3. Results

**Cohort Description and Baseline Characteristics**

Of the initially eligible participants, individuals with incomplete baseline fibrosis assessment or missing key covariate data were excluded from longitudinal modeling. The final analytic cohort comprised 304 participants contributing 1216 person-visits, of which 421 (34.6%) met criteria for a visit-level fibrosis risk event. Baseline fibrosis indices were largely non-advanced, with heterogeneous metabolic profiles.

**Baseline vs. Longitudinal Comparisons**

Baseline metabolic markers demonstrated weak cross-sectional correlations with fibrosis. At baseline, mean HOMA-IR did not differ between patients who later experienced fibrosis progression and those who did not (3.04 vs. 3.18; descriptive comparison). In contrast, longitudinal analyses accounting for within-individual variation and time-updated covariates identified stronger associations. Specifically, individuals with fibrosis progression exhibited significantly greater longitudinal increases in HOMA-IR (*p* < 0.01), whereas those without progression maintained relatively stable values throughout follow-up. This divergence between baseline and trajectory comparisons reflects dynamic metabolic-fibrotic relationships not captured in static summaries.

**Incidence of Metabolic Dysfunction**

Among 113 metabolically normal participants at baseline, incident clustered metabolic dysfunction occurred at 21.43 per 100 person-years (95% CI: 14.75–30.09). Person-time was calculated from baseline until the last recorded visit or fibrosis progression event. Baseline demographic and virological factors did not independently predict incident metabolic dysfunction. Missing visit-level data were primarily due to incomplete laboratory panels and were modest in proportion, without apparent systematic variation across fibrosis categories on descriptive assessment.

**Insulin Resistance and Visit-Level Fibrosis Risk Status**

Median baseline HOMA-IR values were comparable across fibrosis strata. However, as noted above, individuals with visit-level fibrosis risk events exhibited significantly greater longitudinal increases in HOMA-IR (*p* < 0.01). Time-updated HOMA-IR remained independently associated with fibrosis progression status at the same visit after adjustment for age, sex, baseline fibrosis, antiviral therapy, and HBV DNA. ALT and AST trajectories did not independently distinguish progression patterns (all *p* > 0.10), and no significant interactions were observed between HOMA-IR and antiviral therapy or HBeAg status. Table 1 presents the longitudinal trajectories of metabolic indices and liver biochemistry across the four timepoints.

**Other Metabolic Components**

Other metabolic components showed limited independent contributions. Triglycerides demonstrated weak unadjusted associations but were not significant after adjustment, and BMI showed no independent association. Table 2 presents the adjusted longitudinal associations between time-updated predictors and fibrosis progression.

**Time-Lag Sensitivity Analysis (Supplementary Materials)**

A time-lag sensitivity analysis was conducted to explore whether insulin resistance measured at the preceding visit was associated with fibrosis risk status at the subsequent visit. Using generalized estimating equations (GEE) with HOMA-IR measured at the preceding visit (t − 1, six months prior) as a predictor of fibrosis status at the current visit (t), the analysis yielded an attenuated but directionally consistent association (OR = 1.028, 95% CI: 1.015–1.041, *p* < 0.001) compared to the primary contemporaneous association (OR = 1.041, 95% CI: 1.030–1.052).

**Clinical Utility Model: Mechanistic vs. Pragmatic Distinction**

Two distinct models were developed in this study, serving complementary purposes:

Mechanistic Model (Table 2): This model includes HOMA-IR as the primary exposure and evaluates its independent association with visit-level fibrosis risk status, adjusting for covariates (age, sex, baseline fibrosis, antiviral therapy, HBV DNA). HOMA-IR requires fasting insulin measurement, which is not routinely available in all clinical settings. This model addresses the mechanistic objective of understanding whether insulin resistance independently associates with fibrosis status.

**Pragmatic Clinical Utility Model (Table 3)**: This model was developed for resource-limited settings where insulin assays may be unavailable. It incorporates routinely available parameters: age, fasting blood glucose (as a proxy for metabolic dysregulation), AST, platelet count, and triglycerides. HOMA-IR is excluded from this model by design to maximize accessibility. The model demonstrated moderate discrimination (AUC = 0.772). Grouped 5-fold cross-validation yielded a pooled AUC of 0.766 (mean ± SD: 0.766 ± 0.027), indicating stable performance across folds. Bootstrap validation with 2000 resamples estimated minimal optimism (0.004), resulting in an optimism-corrected AUC of 0.768. The events-per-variable ratio was 84.2 (421 events across 5 predictors), indicating low risk of overfitting.

Calibration analysis showed a slope of 1.00 and an intercept of 0.10, indicating minimal overfitting and limited systematic bias. The Brier score was 0.168. Visual inspection of the calibration plot (Supplementary Figure S3) demonstrated close agreement between predicted and observed risks across most probability ranges, with minor deviation in lower-risk strata

**Risk Stratification**

Table 4 presents the pragmatic clinical utility model’s performance across predicted probability-based risk strata.

Observed event rates increased across predicted strata, supporting effective relative risk stratification. However, absolute probabilities were underestimated in lower-risk groups, indicating imperfect calibration. The low-risk stratum comprised 41 visits, and the corresponding estimates should be interpreted cautiously given the limited sample size.

## 4. Discussion

This longitudinal analysis identifies insulin resistance (IR) as the most consistent metabolic correlate of fibrosis status in chronic HBV. Unlike transaminases, which primarily reflect fluctuating hepatocellular injury, time-updated IR remained independently associated with visit-level fibrosis risk status after accounting for inflammatory activity [16,17]. These findings highlight the importance of the metabolic state as a parallel biological axis that influences fibrogenic processes.

Mechanistically, IR may promote hepatic stellate cell activation and extracellular matrix deposition, providing biological plausibility for its association with fibrosis risk, although the present study cannot establish causal directionality [9,18,19]. In contrast to non-alcoholic fatty liver disease (NAFLD), BMI and dyslipidemia were not independent predictors in this cohort [9,20,21,22]. Some HBV studies have reported steatosis coexisting with less severe fibrosis after adjustment for viral factors, suggesting that systemic adiposity may not adequately capture intrahepatic metabolic stress in viral hepatitis [8,23]. These findings support the presence of disease-specific metabolic phenotypes in HBV, in which insulin signaling pathways may exert a greater influence on fibrogenesis than conventional anthropometric or lipid markers.

The derived clinical utility model demonstrated moderate discrimination using routinely available parameters (AUC = 0.772), with stable performance on grouped cross-validation (0.766 ± 0.027) and minimal optimism after bootstrap correction (0.768). While higher-performing models have been reported in HBV and broader populations, these often incorporate imaging or expanded biomarker panels [24,25,26,27]. In contrast, the present framework prioritizes accessibility and interpretability, enabling visit-level risk stratification in settings where elastography is unavailable.

A time-lag sensitivity analysis was conducted to explore the temporal directionality of the association between insulin resistance and fibrosis progression. Using HOMA-IR measured at the preceding visit (t − 1, six months prior) as a predictor of fibrosis status at the current visit (t), the analysis yielded an attenuated but directionally consistent association (OR = 1.028, 95% CI: 1.015–1.041, *p* < 0.001) compared to the primary contemporaneous association (OR = 1.041, 95% CI: 1.030–1.052). This attenuation is expected given metabolic variability over 6-month intervals. Importantly, these findings support longitudinal co-variation but do not establish causal directionality. These time-lag findings are consistent with published evidence examining metabolic factors in CHB [28,29].

### 4.1. Limitations

Several design constraints warrant consideration. First, metabolic exposures and fibrosis status were analyzed contemporaneously at the same visit, without a time-lag structure; accordingly, observed associations reflect time-resolved co-variation rather than temporally antecedent or predictive effects. The time-lag sensitivity analysis provided directionally consistent but attenuated estimates, which support longitudinal association but do not establish causation. Second, the hierarchical use of non-invasive fibrosis indices (APRI and FIB-4), which incorporate age, AST, and platelet count, introduces partial mathematical coupling with predictors included in the model (age, AST, platelets); this overlap can inflate measures of association, and the model is therefore specified as a pragmatic clinical triage framework rather than an estimator of independent structural fibrosis progression. Third, patients with incomplete longitudinal records or irregular follow-up were excluded during cohort construction, resulting in a final analytical dataset with no missing values; while this ensures internal consistency for repeated-measures analyses, it may introduce selection bias if missingness is associated with metabolic severity or follow-up patterns, as these patients may differ systematically in disease severity, healthcare access, or metabolic risk profile, potentially limiting generalizability. Additional limitations include the observational design, which precludes causal inference; residual confounding from unmeasured factors (lifestyle, diet, genetic predispositions); and a single-center, geographically restricted cohort, which limits generalizability to broader HBV populations. External validation in independent cohorts, ideally incorporating imaging-defined outcomes, is required to confirm generalizability.

### 4.2. Strengths

Key strengths include the use of repeated, time-updated metabolic exposures across 1216 person-visits, the application of GEE and mixed-effects approaches to account for within-patient correlation, and the use of grouped cross-validation and bootstrap validation to reduce optimism in model performance. The additional time-lag sensitivity analysis further strengthens the interpretation by showing directionally consistent associations when prior-visit HOMA-IR was related to subsequent fibrosis risk status. The pragmatic model also emphasizes clinical applicability by relying on routinely available variables in settings where advanced fibrosis assessment may be limited.

## 5. Conclusions

In adults with chronic HBV, insulin resistance is consistently linked to current fibrosis status and appears to perform better than traditional metabolic markers. Looking at changes over time offers more biological insight than a single baseline assessment, although time-lagged analyses are still needed to better understand the direction of this relationship. A visit-level clinical utility model based on routinely available parameters showed moderate discriminative ability for practical risk stratification. Although some of its performance may be due to overlap between the predictors and non-invasive fibrosis measures, the model emphasizes accessibility and real-world usefulness over strict mechanistic separation. Incorporating longitudinal metabolic monitoring into HBV care may help identify patients at risk of progressive fibrosis earlier, especially in resource-limited settings. Overall, these findings support adding time-sensitive metabolic signals to fibrosis risk frameworks, moving beyond models based only on virological and static biochemical measures. External validation in independent cohorts is still needed to confirm generalizability.

## Figures and Tables

**Table 1:** Longitudinal trajectories of metabolic indices and liver biochemistry.

Parameter	Baseline	6 Months	12 Months	18 Months
HOMA-IR, mean ± SD	3.16 ± 1.37	3.20 ± 1.40	3.24 ± 1.42	3.26 ± 1.61
Fasting glucose (mg/dL)	106.3 ± 17.6	106.7 ± 17.9	106.7 ± 18.0	108.3 ± 18.6
ALT (U/L)	32.5 ± 15.6	33.1 ± 16.4	33.4 ± 17.2	34.3 ± 19.1
AST (U/L)	27.6 ± 12.7	28.0 ± 13.0	28.2 ± 13.7	28.8 ± 15.6
Platelets (×10^3^/µL)	223 ± 51	223 ± 52	222 ± 53	221 ± 55

Footnote: Visit-level summaries across 1216 observations.

**Table 2:** Longitudinal associations with visit-level fibrosis risk status.

Predictor (Time-Updated)	Adjusted OR	95% CI	*p*-Value
HOMA-IR	1.04	1.03–1.05	<0.001
BMI	1.02	0.98–1.06	0.460
Triglycerides	1.002	1.000–1.004	0.102
Fasting glucose	1.014	1.005–1.023	0.002
AST	1.083	1.068–1.098	<0.001
Platelets	0.991	0.988–0.995	<0.001
Age	1.038	1.024–1.053	<0.001

**Table 3:** Multivariable clinical utility model.

Variable	β	OR	95% CI	*p*-Value
Age	0.0376	1.038	1.024–1.053	<0.001
Fasting glucose	0.0137	1.014	1.005–1.023	0.002
AST	0.0797	1.083	1.068–1.098	<0.001
Platelets	−0.0086	0.991	0.988–0.995	<0.001
Triglycerides	0.0016	1.002	1.000–1.004	0.102

**Table 4:** Model performance across risk strata.

Risk Group	Predicted Probability	Visits	% Cohort	Observed Fibrosis Rate
Low	<5%	41	3.4%	17.1%
Moderate	5–15%	239	19.7%	15.1%
High	15–30%	321	26.4%	20.2%
Very high	>30%	615	50.6%	50.9%

## Data Availability

De-identified participant data are available from the corresponding author upon reasonable request and subject to institutional approval. Statistical analysis code is available upon request to facilitate reproducibility.

## Supplementary Materials

The following supporting information can be downloaded at: https://www.mdpi.com/article/10.3390/life16060939/s1. Figure S1: Cumulative Incidence of Metabolic Dysfunction in Chronic Hepatitis B Patients; Figure S2: Receiver Operating Characteristic Curve for the Visit-Level Fibrosis Risk Stratification Model; Figure S3: Calibration Curve of Predicted vs. Observed Fibrosis; Table S1. Methodological comparison of studies examining metabolic factors and fibrosis in chronic hepatitis B; Table S2: Baseline demographic, virological, metabolic, and liver-related characteristics of patients with chronic hepatitis B (*n* = 304); Table S3: Repeated-measures correlation matrix of metabolic variables and fibrosis indices. Table S4: Time Lag Results. References [4,5,30,31,32,33,34,35] are cited in the Supplementary Materials.
