# Supplementary material for: Insulin Resistance as a Dynamic Correlate of Fibrosis Status in Chronic Hepatitis B: A Visit-Level Longitudinal Risk Stratification Framework
Source: Life (Basel). 2026 Jun 2;16(6):939. doi: 10.3390/life16060939 (PMC13301149; doi:10.3390/life16060939)
Supplement: Supplementary file 1 [file life-16-00939-s001.zip › life-4249828-supplementary.pdf]

Supplementary Materials

Table S1. Methodological comparison of studies examining metabolic factors and fibrosis in chronic hepatitis B

Study (year)	Design	Sample	Metabolic measure(s)	Fibrosis assessment	Repeated measures	Time-updated	Key finding on IR
Ye et al. (2019) [5]	Cross-sectional	970 CHB	HOMA-IR	Biopsy/APRI	No	No	IR more prevalent in CHB+NAFLD
Kaya et al. (2021) [30]	Cross-sectional	94 CHB	HOMA-IR	FIB-4	No	No	Higher HOMA-IR in CHB vs controls
Mousa et al. (2018) [31]	Cross-sectional	190 CHB	HOMA-IR, leptin	METAVIR	No	No	HOMA-IR and leptin independent predictors of fibrosis
Liu TW et al. (2020) [32]	Retrospective cohort	2,402 CHB	BMI	Age-adjusted FIB-4	Yes (serial)	No (baseline BMI only)	Overweight associated with slower FIB-4 increase
Huang et al. (2025) [4]	Retrospective multi-center	7,495 CHB	MetS components	FIB-4, elastography	No (baseline only)	No	Metabolic disease lowers treatment response rates
Huang SC et al. (2024) [33]	Retrospective cohort	11,502 CHB	MD criteria (5 components)	Clinical outcomes	No	No	MD increases cirrhosis risk (aHR 1.82)
Zheng et al. (2021) [34]	Meta-analysis	32 studies (14,435 CHB)	BMI, steatosis	Biopsy, non-invasive	No	No	Steatosis not consistently predictive of fibrosis
Liu L et al. (2025) [35]	Retrospective cross-sectional	502 CHB	Metabolic score	APRI, FIB-4	No	No	Metabolic score predicted fibrosis
**Present study**	**Retrospective longitudinal**	**304 CHB (1,216 visits)**	**HOMA-IR, BMI, lipids (time-updated)**	**APRI, FIB-4, imaging**	**Yes (4 timepoints)**	**Yes (visit-level)**	**Time-updated HOMA-IR associated with fibrosis status (OR 1.04, p<0.001)**

Table S2. Baseline demographic, virological, metabolic, and liver-related characteristics of patients with chronic hepatitis B (n = 304)

**Characteristic**	**Overall cohort**
Number of patients, n	304
Age, years, mean ± SD	40.24 ± 11.6
Male sex, n (%)	188 (61.8)
BMI, kg/m², mean ± SD	28.58 ± 5.16
HOMA-IR, mean ± SD	3.18 ± 1.41
ALT, U/L, mean	31.9
AST, U/L, mean	27.8
HBeAg positive, n (%)	67 (22.0)
HBeAg negative, n (%)	237 (78.0)
**Baseline liver disease stage, n (%)**	
Cirrhosis (F4)	13 (4.3)
Moderate fibrosis (F2–F3)	26 (8.6)
Mild fibrosis (F1–F2)	3 (1.0)
Fatty liver	6 (2.0)
Hepatocellular carcinoma	2 (0.7)
Not indicated	254 (83.6)

Footnote:
Baseline fibrosis categories were defined using imaging and/or clinical records. HOMA-IR was calculated using fasting glucose and insulin.

Table S3. Repeated-measures correlation matrix of metabolic variables and fibrosis indices

**Variable pair**	**r**	**p-value**
HDL vs FIB-4	−0.070	0.035
HDL vs APRI	−0.091	0.006
Triglycerides vs ALT	0.088	0.008
Total cholesterol vs FIB-4	−0.079	0.017
HOMA-IR vs FIB-4	0.020	0.612

Table S4: Time-lag results

Analysis	OR	95% CI	p-value
Contemporaneous (HOMA-IR at t)	1.041	1.030–1.052	<0.001
Time-lag association (HOMA-IR at t-1 → fibrosis risk status at t)	1.028	1.015–1.041	<0.001

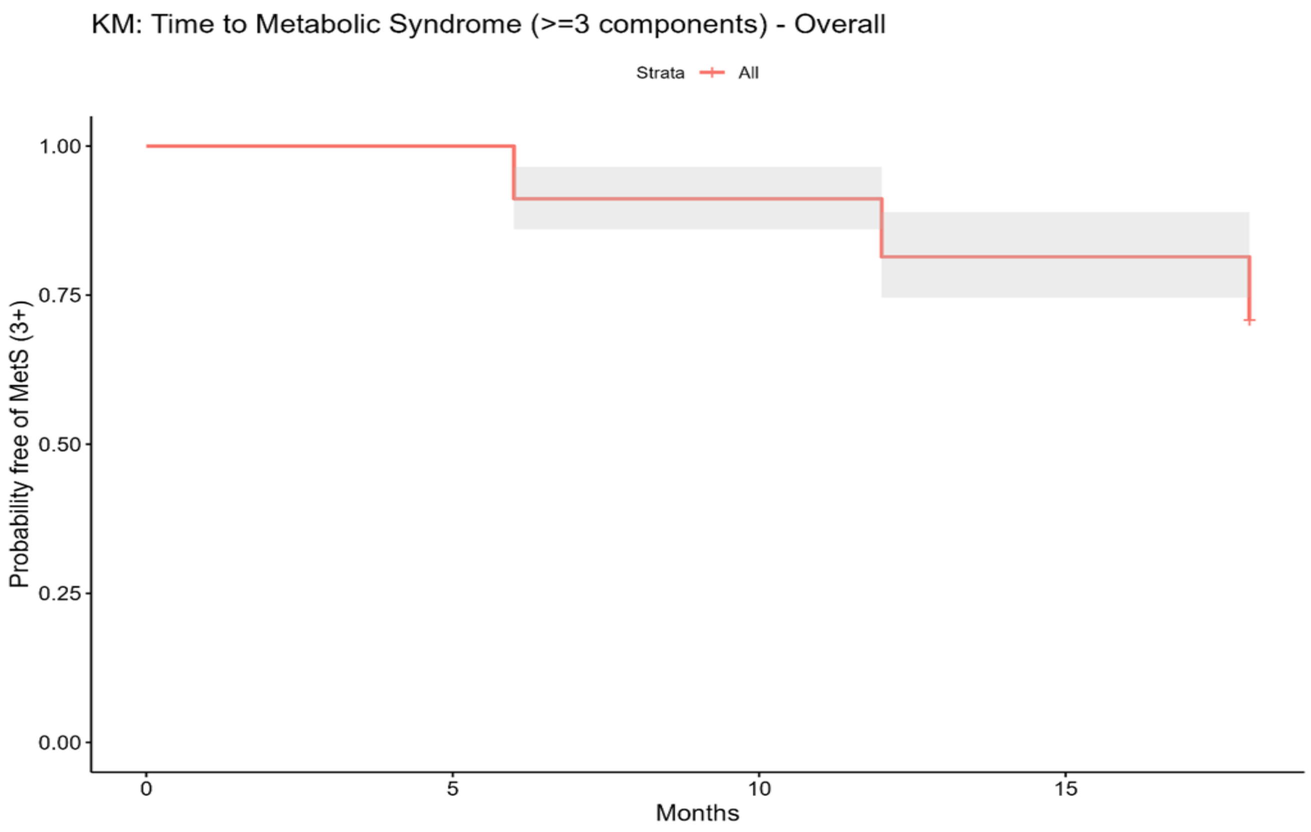

Figure S1. Cumulative Incidence of Metabolic Dysfunction in Chronic Hepatitis B Patients

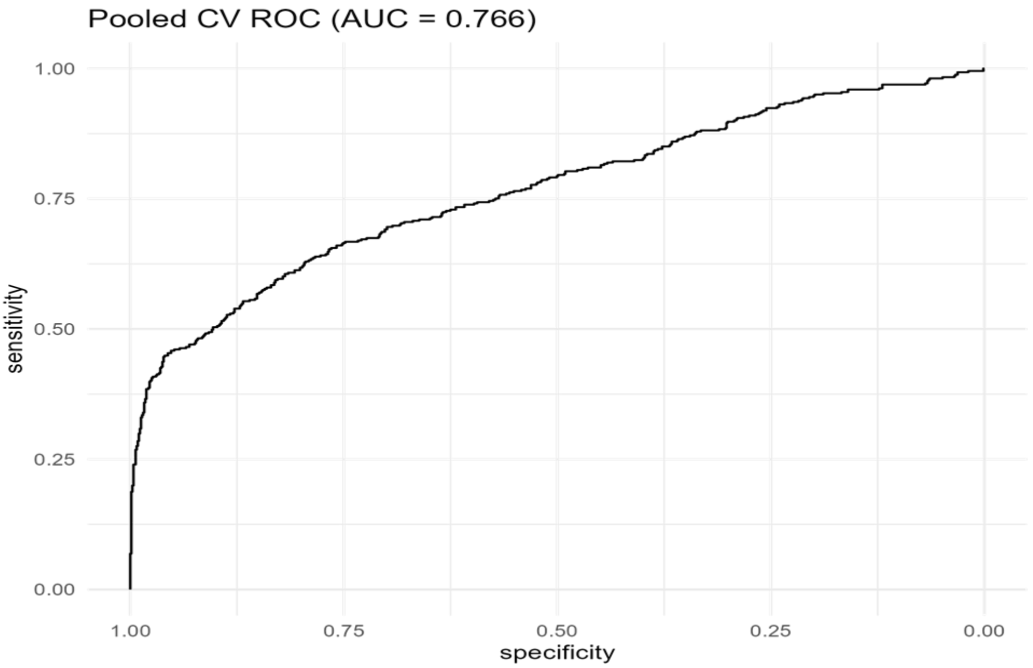

Figure S2. Receiver Operating Characteristic Curve for the Visit-Level Fibrosis Risk Stratification Model

ROC curve demonstrating the discriminative performance of the multivariable model for classifying visit-level fibrosis risk status. The AUC was 0.772 in the apparent dataset and 0.766 after internal cross-validation, indicating moderate discrimination. The model incorporates age, AST, platelet count, and fasting blood glucose and should be interpreted as a pragmatic risk stratification framework rather than a causal prediction model.

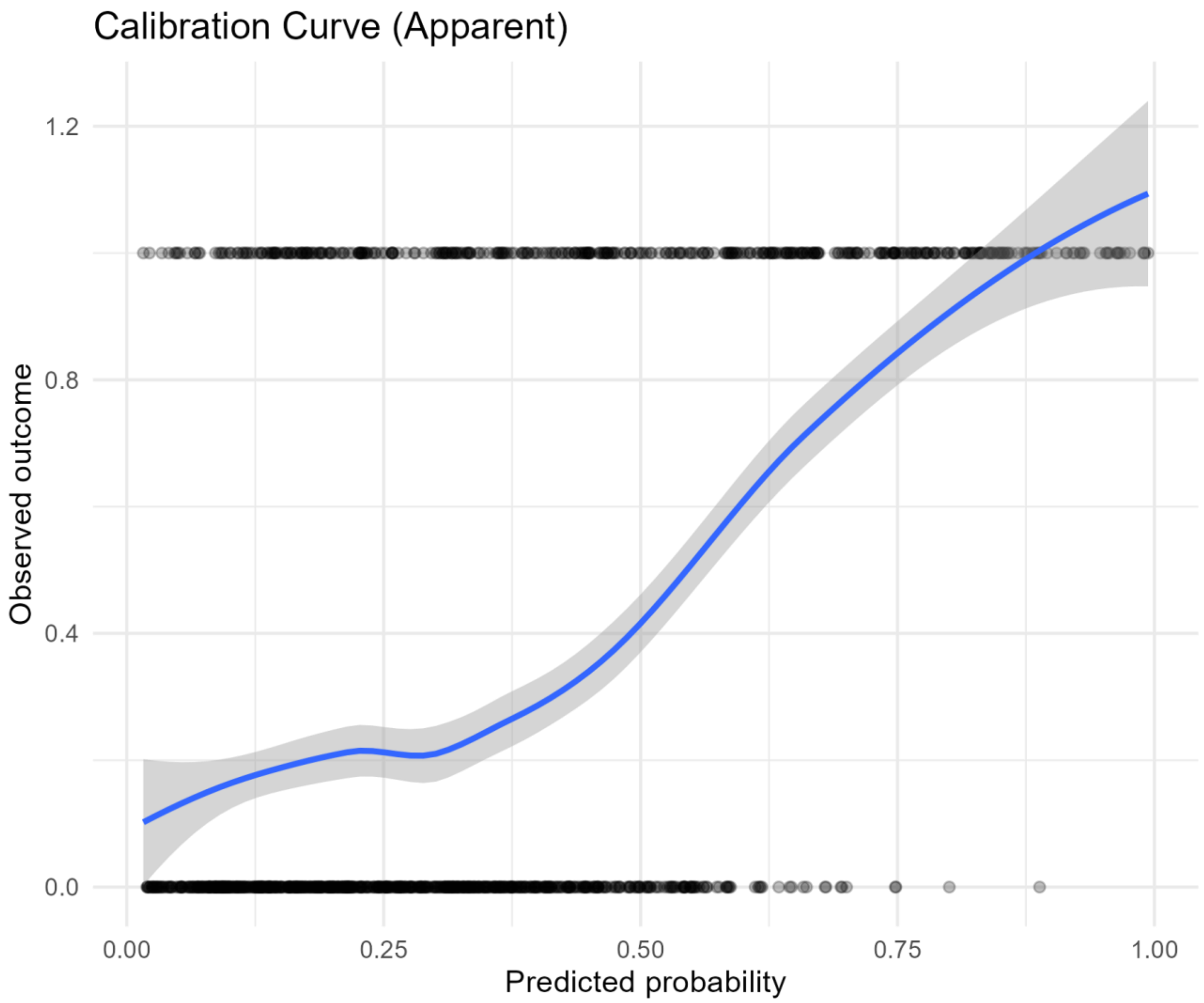

Figure S3: Calibration Curve of Predicted vs. Observed Fibrosis
